# Increase methylmercury accumulation in *Arabidopsis thaliana* expressing bacterial broad-spectrum mercury transporter MerE

**DOI:** 10.1186/2191-0855-3-52

**Published:** 2013-09-03

**Authors:** Yuka Sone, Ryosuke Nakamura, Hidemitsu Pan-Hou, Masa H Sato, Tomoo Itoh, Masako Kiyono

**Affiliations:** 1 Department of Public Health and Molecular Toxicology, School of Pharmacy, Kitasato University, 5-9-1 Shirokane, 108-8641 Minato-ku, Tokyo, Japan; 2 Faculty of Pharmaceutical Sciences, Setsunan University, 45-1 Nagaotogecho, 573-0101 Hirakata, Osaka, Japan; 3 Graduate School of Life and Environmental Sciences, Kyoto Prefectural University, 1-5, Shimogamonakaragi-cho, 606-8522 Sakyo-ku, Kyoto, Japan

**Keywords:** Bacterial broad-spectrum mercury transport, MerE, Methylmercury, Phytoremediation

## Abstract

The bacterial *merE* gene derived from the Tn*21 mer* operon encodes a broad-spectrum mercury transporter that governs the transport of methylmercury and mercuric ions across bacterial cytoplasmic membranes, and this gene is a potential molecular tool for improving the efficiency of methylmercury phytoremediation. A transgenic *Arabidopsis* engineered to express MerE was constructed and the impact of expression of MerE on methylmercury accumulation was evaluated. The subcellular localization of transiently expressed GFP-tagged MerE was examined in *Arabidopsis* suspension-cultured cells. The GFP-MerE was found to localize to the plasma membrane and cytosol. The transgenic *Arabidopsis* expressing MerE accumulated significantly more methymercury and mercuric ions into plants than the wild-type *Arabidopsis* did. The transgenic plants expressing MerE was significantly more resistant to mercuric ions, but only showed more resistant to methylmercury compared with the wild type *Arabidopsis.* These results demonstrated that expression of the bacterial mercury transporter MerE promoted the transport and accumulation of methylmercury in transgenic *Arabidopsis*, which may be a useful method for improving plants to facilitate the phytoremediation of methylmercury pollution.

## Introduction

Mercury pollution is still a worldwide problem in environments because of natural events and human activities such as coal burning, industrial use and gold-mining activities (Harada 1995). Metallic and ionic form of mercury can accumulate in sediments, where they are readily converted to highly toxic methylmercury by microbes (Barkay et al. 2003). Clinical investigations have shown that methylmercury is the principal form of mercury that accumulated in fish and biomagnifies in their consumers, causing severe neurodegenerative symptoms (Harada 1995). The severe adverse effects of this contaminant mean there is an urgent need to develop an effective and affordable technology to facilitate its removal from the environment.

Phytoremediation refers to the use of green plants in the removal of environmental pollutants, which is recognized as a cost effective, sustainable, and environmentally friendly approach that has many advantages during the large-scale clean-up of contaminated sites (Clemens et al. 2002; Kramer 2005; Malik 2004; Ruiz and Daniell 2009; Salt 1998). In recent studies, Meager et al. engineered bacterial *mer* operons such as MerA (mercuric reductase) to reduce reactive mercuric ions to volatile and relatively inert monoatomic Hg(0) vapor, and MerB (organomercurial lyase) to degrade methylmercury to mercuric ions into plants, thereby remediating methylmercury contamination (Meagher and Heaton 2005). Plants such as cottonwood trees (Lyyra et al. 2007) and tobacco (Heaton et al. 2003) have been modified to express either MerB or both MerB and MerA, which convert methylmercury to mercuric ions or mercury vapor, respectively. The disadvantage of this approach is that elemental mercury Hg(0) is released into the environment, where it accumulates and can eventually be converted into highly toxic methylmercury. To help address this environmental problem, a new methylmercury remediation method is required to replace the *merA-*mediated mercury reduction mechanism so plant cells can accumulate methylmercury from contaminated sites without releasing mercury vapor into the ambient air.

In general, rehabilitation of metal-contaminated soils by plants requires a long time for the purification process to be completed. McGrath and Zhao reported that several months were required to reduce the mercury content by half in contaminated soils (McGrath and Zhao 2003). The expression of mercury transporter in the plant may provide a means of improving mercury uptake, thereby shortening the purification completion time. In a previous study, we demonstrated for the first time that the MerE protein encoded by pE4 is localized in the membrane cell fraction and that MerE is a novel, broad-spectrum mercury transporter, which governs the transport of CH_3_Hg(I) and Hg(II) across bacterial cytoplasmic membranes (Kiyono et al. 2009; Sone et al. 2010).

The current study evaluated the feasibility of engineering transgenic *Arabidopsis* plants to express bacterial broad-spectrum mercury transporter, MerE and its potential use in the phytoremediation of methylmercury pollution. This study showed that expression of MerE promoted the transport and accumulation of methylmercury in transgenic *Arabidopsis.* Enhanced methylmercury accumulation mediated by MerE represents one way for improving plants to be more suitable for use in phytoremediation of methylmercury pollution.

## Materials and methods

### Materials and growth conditions

Table 1 shows the *Escherichia coli* (*E. coli*), *Arabidopsis thaliana (A. thaliana) -*cultured cells and plasmids used in this study. *E. coli* XL1-Blue was grown at 37°C in Luria-Bertani (LB) medium and used for routine plasmid propagation. When necessary, the medium was supplemented with 25 μg/mL kanamycin. Suspension-cultured *A. thaliana* cells were maintained in Murashige and Skoog (MS) medium on a rotary shaker at 22°C with continuous white light and sub-cultured once a week. *A. thaliana* ecotype Columbia was used for the plant transformations. The seeds produced were germinated and grown on jiffy-7 peat pellets, whereas the surface-sterilized seeds were sown onto MS agar plates. The plants were grown in an environmental growth chamber (Sanyo, Tokyo, Japan) at 22°C in long-day (16 h light/8 h dark) conditions.

**Table 1:** Strains and plasmids used in this study

**Strains and plasmids**	**Description of relevant features(s)**	**Reference or source**
Strains		
*E.coli* XL1-Blue	*recaAI endAI gyrA96 thi hsdR17 supE44 reIAI lac/[F::Tn/0proAB + lac1q lacZM15 traD36*	(Bullock et al. 1987)
*Arabidopsis thaliana* suspension-cultured cells	Alex	(Ueda et al. 2001)
Plasmids		
pE4	*meR-o/p-merE* in pKF19k	(Kiyono et al. 2009)
pMAT137	None: binary expression vector with a CaMV35S promoter produced by tandem duplication of the enhancer sequence	(Matsuoka and Nakamura 1991)
CaMV35S(S65T)-NOS3′	GFP in pUC18	(Uemura et al. 2004)
pE18	GFP-MerE in pUC18	This study
pMAE2	MerE in pMAT137	This study

### Enzymes and reagents

The restriction enzymes, the DNA ligation kit and Taq polymerase were obtained from Takara Shuzo Corp. (Kyoto, Japan). Analytical reagent grade mercury was purchased from Wako Chemicals (Tokyo, Japan).

### Plasmid construction

The recombinant plasmids and oligonucleotide primers used in this study are described in Table 1 and Table 2, respectively. The plasmid pE18 that carried the *gfp-merE* fusion gene was constructed in pUC18 as follows. A 0.23 kb fragment containing *merE* was PCR-amplified using the primers U-Bgl-merE and L-Kpn-merE, which contained *Bgl*II and *Kpn*I sites. The pE4 plasmid (Kiyono et al. 2009) was used as a template. After digestion the PCR product with *Bgl*II and *Kpn*I, the fragment was cloned into the corresponding sites in CaMV35S-sGFP(S65T)-NOS3′ (Uemura et al. 2004) and sequenced, and then designated as pE18.

**Table 2:** Oligonucleotide primers used in this study

**Primer**	**Oligonucleotide (5′ → 3′)**
U-Bgl-merE	GAAGATCTATGAACGCCCCTGACAAA
L-Kpn-merE	GGGGTACCTCATGATCCGCCCCGGAA
U-Not-merE	AAGGAAAAAAGCGGCCGCATGAACGCCCCTGACAAACT
L-Xba-merE	GCTCTAGATCATGATCCGCCCCGGAAGG
U-321NPTII	ATTGAACAAGATGGATTGCA
L-1109NPTII	GAAGAACTCGTCAAGAAGGC
β- ACT-Fd	CAACTGGGACGACATGGAGA
β- ACT-Rv	GATCCACATCTGCTGGAAGG

The plasmid pMAE2 that carried the *merE* gene was constructed in pMAT137 (Matsuoka and Nakamura 1991) as follows. The primers U-Not-merE and L-Xba-merE were used to amplify the *merE* region (0.23 kb) with pE18 as a template. After digestion the PCR products with *Not*I-*Xba*I, the fragment was cloned into the *Not*I-*Xba*I sites of pMAT137. The cloned fragment was sequenced and the resulting plasmid was designated as pMAE2.

### Confocal laser scanning microscopy

GFP-fused proteins were transiently expressed in *A. thaliana* suspension-cultured cells using a published method (Uemura et al. 2004). Cells transformed with pE18 were viewed without fixation under an Olympus BX60 fluorescence microscope, which was equipped with a Model CSU10 confocal scanner (Yokogawa Electric) (Nakano 2002) and a Zeiss LSM510 META or LSM5 PASCAL microscope, which were equipped with green HeNe and argon lasers.

### Transformation of plants and confirmation of transgenic plants

The pMAE2 plasmid was introduced into *Agrobacterium tumefaciens* (*A. tumefaciens*) via electroporation (Mozo and Hooykaas 1991). *A. tumefaciens* was grown at 25–28°C in LB medium added with 25 μg/mL kanamycin and employed for the transformation of *A. thaliana. A. thaliana* ecotype Columbia plants were transformed using the floral dip method (Clough and Bent 1998) by Inplanta Innovations Inc. (Kanagawa, Japan). The progeny seedlings were selected on MS medium containing 50 mg/L kanamycin. The third generations of *merE* transgenic plants (T_3_) were used for all experiments described in this paper.

Plant genomic DNA was isolated from transformed and untransformed leaves using the FTA Kit (GE Healthcare, Buckinghamshire, England) in accordance with the manufacturer’s instructions. The target genome DNA was amplified using specific primers, i.e., U-Not-merE and L-Xba-merE for *merE,* according to the manufacturer’s instructions (Table 2). The primers U-321NPTII and L-1109NPTII were used to amplify *NOS-NPTII* in each PCR reaction, as control (Table 2). The PCR products were separated on 2% agarose gels and visualized by ethidium bromide staining.

### Quantitation of mRNA levels by reverse transcription PCR

Total RNA was extracted from cells using an RNeasy Plant Mini Kit (Qiagen, CA, USA), according to the manufacturer’s instructions. The Superscript First-strand Synthesis System for reverse transcription PCR (Life Technologies, CA, USA) was used to prepare single-stranded cDNA. The target cDNAs were amplified using specific primers, i.e., U-Not-merE and L-Xba-merE to *merE,* according to the manufacturer’s instructions. The primers β-ACT-Fd and β-ACT-Rv were used to amplify *ACT1* in each PCR reaction as controls (Table 2). The PCR products were separated on 2% agarose gels and visualized by ethidium bromide staining.

### Subcellular fractionation of *Arabidopsis* tissues

To generate roots, surface-sterilized *A. thaliana* seeds were germinated in sterile MS liquid medium with 100 rpm shaking using a rotary shaker in dark conditions. The roots of 14-day-old plants were homogenized in a grinding buffer, which contained 50 mM Tris–HCl, pH 7.5, 250 mM sorbitol, 5 mM EDTA, 5 mM EGTA, 1 mM dithiothreitol, and 100 μM p-(amidinophenyl) methanesulfonyl fluoride hydrochloride (APMSF). The homogenate was filtered through four layers of Miracloth (EMD Biosciences, Darmstadt, Germany), and centrifuged at 10,000 × *g* for 10 min. The supernatant was centrifuged at 100,000 × *g* for 30 min and the precipitate was then suspended in the grinding buffer.

### SDS-PAGE and immunoblotting

Proteins were separated by SDS-PAGE and transferred to an Immobilon-P membrane (Millipore, Billerica, USA). After blocking with de-fatted milk, the membrane filter was incubated with rabbit anti-MerE polyclonal antibody (Kiyono et al. 2009). The membranes were washed and reacted with peroxidase-conjugated anti-rabbit IgG antibody (Sigma Aldrich, MO, USA). Anti-MerE polyclonal and peroxidase-conjugated anti-rabbit IgG antibodies were used at a dilution of 1:3,000. Chemiluminescent reagents ECL (GE Healthcare, Chalfont St Giles, UK) were used to detect antigens.

### Mercury accumulation

T_3_ transgenic plants were cultured in MS gellan gum plates with different concentrations of HgCl_2_ or CH_3_HgCl for 2 weeks at 22°C. After treatment with 10 μM HgCl_2_ or 0.3 μM CH_3_HgCl, the total amount of mercury accumulated by an entire plant was determined as follows, using 60 plants in total. Entire plants samples were digested with a concentrated acid mixture (nitric acid: perchlonic acid = 4: 1) for 4 h at 90°C and their total cellular mercury contents were measured using an atomic absorption spectrometry analyzer HG-310 (Hiranuma, Japan). The standard deviation of the measurements was less than 10%.

### Mercury resistance

The sensitivities of T_3_ transgenic plants to mercury were tested using the following method. The sterilized seeds from wild-type and transgenic plants were aligned in a horizontal array of MS gellan gum plates, which contained 5 μM HgCl_2_ or 0.3 μM CH_3_HgCl, where they the seeds germinated and grew vertically. The root lengths of the seedlings were measured after 2 weeks’ cultivation at 22°C.

### Statistical analysis

Data analysis was performed using the statistical tools (Student’s t-test) of Microsoft Excel software.

## Results

### Cellular localizations of GFP-MerE in suspension-cultured plant cells

**Figure 1:**
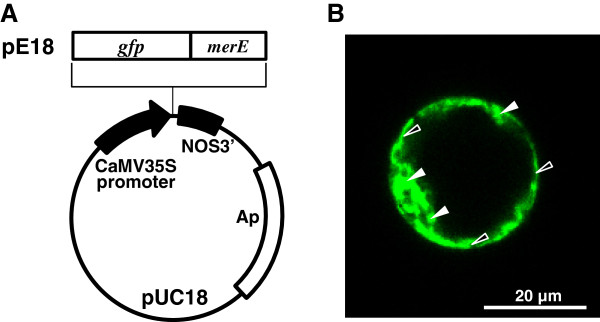
**Construction of *gfp-merE *fusion plasmid (A) and cellular localization of GFP-MerE in suspension-cultured plant cells (B).** GFP-tagged fusion proteins were expressed in suspension-cultured plant cells and viewed by confocal laser scanning microscopy. GFP fluorescence images are shown for GFP-MerE **(B)**. Bars = 20 μm.

To determine the subcellular localization of GFP-fusion proteins in suspension-cultured plant cells, GFP-tagged MerE was expressed transiently (Figure 1A) and its fluorescence was visualized by confocal laser scanning microscopy (Figure 1B). GFP-MerE was detected primarily in the endoplasmic reticulum (Closed arrowheads in Figure 1B), but some fusion proteins were detected in the plasma membrane (Open arrowheads in Figure 1B).

### Expression of MerE in transgenic plants

**Figure 2:**
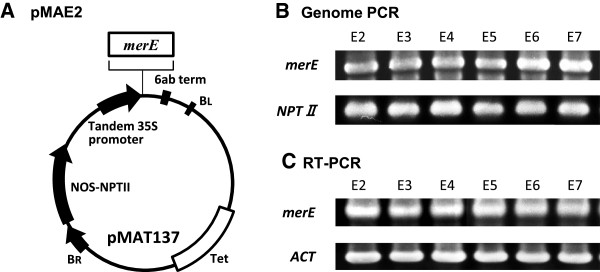
**Characterization of *merE *in transgenic plant lines.** The structure of the DNA region transferred using the plant expression plasmids pMAE2 **(A)**. Confirmation of the expression of *merE* (E2, E3, E4, E5, E6, and E7) in transgenic plants based on genomic PCR expression analysis **(B)**. Expression analyses of *merE* (E2, E3, E4, E5, E6, and E7) in transgenic plants, which were determined by reverse transcription PCR **(C)**. Transgenic plants were grown on MS gellan gum plates. Plates were incubated at 22°C for 2 weeks. Genome DNA prepared from transgenic plants and used for genomic PCR analysis of *merE* (U-Not-merE and L-Xba-merE) and *NPT II* (U-321NPTII and L-1109NPTII). The total RNA was prepared from transgenic plants and used for reverse transcription PCR analysis to determine the *merE* (U-Not-merE and L-Xba-merE) and *Actin* (primer β-ACT-Fd and β-ACT-Rv) transcription levels.

The PCR-amplified *merE* DNA fragment was subcloned into a binary vector, pMAT137, to generate the plasmid pMAE2 (Figure 2A). The plasmid was transformed into *A. thaliana* ecotype Columbia via *Agrobacterium-*mediated gene transfer (Clough and Bent 1998). Eleven *merE* independent transgenic lines were obtained by selection using MS medium containing 50 mg/L kanamycin. The MerE transformants exhibited no distinctive phenotypes. For instance, the MerE transgenic plants had an equally normal growth as wild-type *Arabidopsis.* Six of eleven *merE* transgenic lines (lines E2, E3, E4, E5, E6, and E7) were selected for further studies. To confirm the presence of the *merE* transgene in the transgenic plants, total genomic DNA was extracted from the mature leaves of T_3_ progeny, and the regions of the introduced *merE* fragments were amplified using the genomic DNA as a template. As expected, 0.23 kb PCR fragment was detected in the *merE* (lines E2, E3, E4, E5, E6 and E7) when U-Not-merE/L-Xba-merE were used as PCR primers (Figure 2B). The expression levels of *merE* in plants were determined by reverse transcription-PCR (RT-PCR). The total RNA was isolated from leaves and specific primers were used to detect *merE* and *Actin* mRNA. The *merE* mRNA was detected in all transformants tested (Figure 2C). There was no significant difference in the mRNA levels of the individual transgenic lines, so the E2 transgenic plant was selected for further studies.

**Figure 3:**
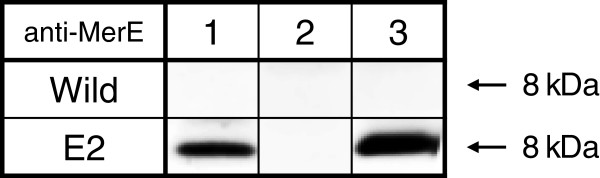
**Immunoblot of MerE protein in transgenic plants.** Immunoblot analysis of the crude cell extracts (lane 1), soluble fractions (lane 2), and membrane fractions (lane 3) obtained from wild type (Wild) and *merE* (E2) transgenic plants, which were performed using anti-MerE polyclonal antibodies. The amount of protein applied in each lane was 25 μg. The arrow indicates MerE (8 kDa).

The expression levels of MerE protein were measured in transgenic plants (lines E2) by immunoblot analysis using a polyclonal anti-MerE antibody, which was prepared in a previous study (Kiyono et al. 2009). A novel protein band of 8 kDa, which reacted specifically with the anti-MerE antibody, was identified in the crude cell extract and crude membrane fraction from transgenic plants (Figure 3, lane 1&3), whereas no protein band reacted with anti-MerE antibody was detected in the soluble fraction (Figure 3, lane 2). The protein size was consistent with the size predicted from the translation of the DNA sequence of the *merE* gene*.* These results demonstrated that the *merE* gene in transgenic plants was transcribed and translated into proteins with molecular mass of 8 kDa, which were probably located in the membrane fraction.

### Effect of MerE expression on mercury accumulation and mercury resistance in transgenic plants

**Figure 4:**
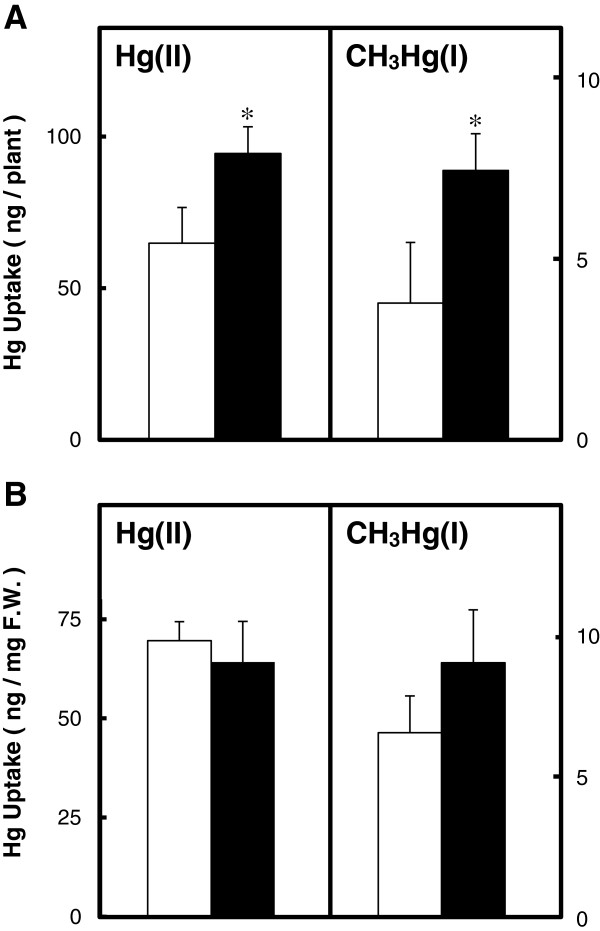
**Accumulation of mercury from MS gellan gum plates containing Hg(II) and CH**_**3**_**Hg(I).** The amounts of mercury (**A**; ng/plant, **B**; ng/fresh weight) that accumulated in wild-type plants (Wild; empty bar) and *merE-* transgenic plants (black bar) were determined after culture for 2 weeks on MS gellan gum plates with various concentrations of HgCl_2_ or CH_3_HgCl, as described in the Materials and Methods. The data are expressed as the means ± S.E.M. based on four determinations from three independent experiments. *P < 0.05 *vs.* the wild type.

Mercury accumulation was determined in transgenic *Arabidopsis* plants after exposure to HgCl_2_ or CH_3_HgCl in MS gellan gum plates for 2 weeks. After treatment with 10 μM HgCl_2_, the amount of mercury accumulated in the entire *merE* transgenic plants were higher than that in wild-type plants (Figure 4A). After treatment with 0.3 μM CH_3_HgCl, the amount of mercury accumulated in the entire *merE* transgenic plants were about 2-fold higher than that in the wild-type plants (Figure 4B).

**Figure 5:**
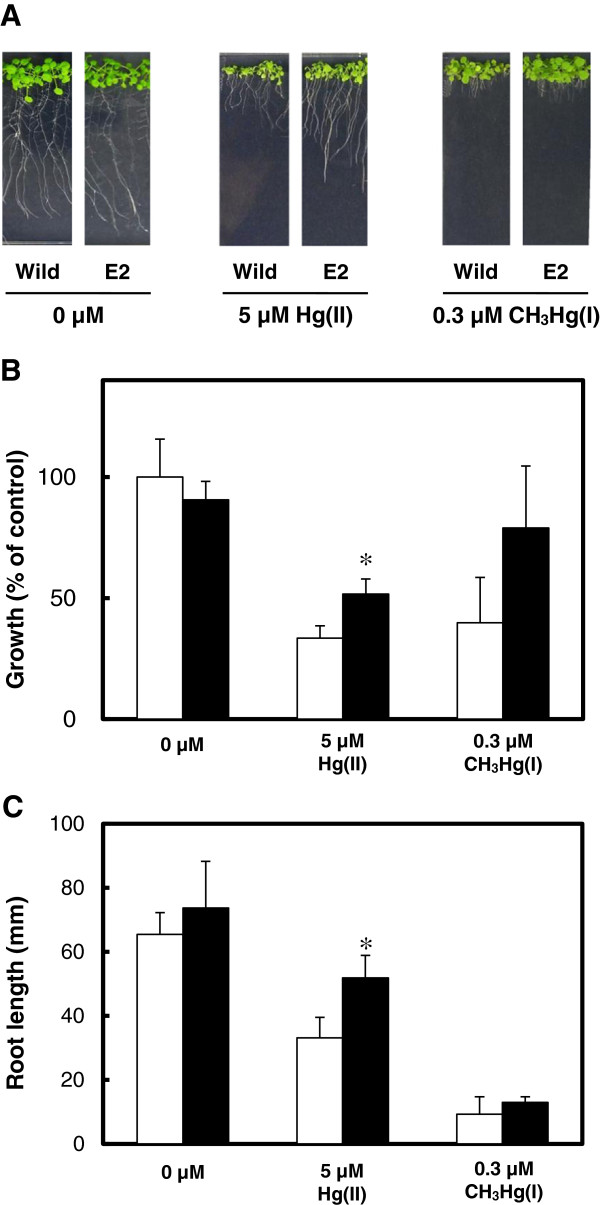
**Susceptibility of transgenic plants to Hg(II) and CH**_**3**_**Hg(I).** Sterilized seeds of wild-type (Wild) and transgenic (E2) plants were germinated and grown on MS gellan gum plates in the presence or absence of 5 μM HgCl_2_ and 0.3 μM CH_3_HgCl **(A)**. After culture for 2 weeks at 22°C, the total wet weights of plants **(B)** and root growth **(C)** levels of wild-type plants (empty bar) and *merE-* transgenic plants (black bar) were evaluated as described in the Materials and Methods. The total wet weight of the wild-type plants in the absence of mercurials was considered as control **(B)**. The data are expressed as the means ± S.E.M. based on four determinations from three independent experiments. *P < 0.05 *vs.* the wild type.

The effect of MerE expression on mercury resistance was evaluated in transgenic *Arabidopsis* plants (lines E2) by monitoring the root and shoot growth of seedlings after exposure to HgCl_2_ or CH_3_HgCl in MS gellan gum plates for 2 weeks. In the absence of mercury, *merE* transgenic plants exhibited the same normal growth as wild-type *Arabidopsis* (Figure 5A). In the presence of 5 μM HgCl_2_ or 0.3 μM CH_3_HgCl, root and shoot growth were inhibited in transgenic seedlings and wild-type *Arabidopsis* (Figure 5A). However, the shoot and root growth of the *merE* transgenic seedlings indicated significant tolerance compared with wild-type *Arabidopsis* in the presence of 5 μM HgCl_2_ (Figure 5A–C). The shoot and root growth of *merE* transgenic seedlings also appeared to be better than that of wild-type *Arabidopsis* in the presence of 0.3 μM CH_3_HgCl (Figure 5A–C).

## Discussion

Phytoremediation, using green plants to remove environmental pollutants including hazardous toxic metals removal from a large volume of contaminated sites is recognized as a cost-effective, sustainable and aesthetically pleasing technology (Tong et al. 2004). However, the use of plants, like all biological methods, does not allow 100% removal of contaminants because the remediation rates decrease as the concentrations of contaminant decrease (Clemens et al. 2002). In addition, phytoremediation is a slow process that requires a long time to complete the purification (McGrath and Zhao 2003). These potential faults may predominantly result from the low metal-uptake activity and thereby limit its usefulness for practical application.

Among the strategies being used to overcome these disadvantages is the use of metal transporter to boost the uptake and transport of metal from soil into transgenic plants (Song et al. 2003). Expression of heavy metal transporter or periplasmic Hg(II)-binding protein genes under the control of a constitutive or inducible promoter may provide a means of improving metal uptake, thereby shortening the phytoremediation completion time (Kiyono et al. 2013; Kiyono et al. 2012; Nagata et al. 2009; Hsieh et al. 2006). In the present study, a transgenic *Arabidopsis* plants engineered to express mercury transporter, MerE (Kiyono et al. 2009; Sone et al. 2010) was constructed and the impact of expression of MerE on methylmercury accumulation was evaluated.

By using the *Agrobacterium-*floral dip method, many independent transgenic *Arabidopsis* plants were obtained. The results obtained by genomic PCR (Figure 2B), RT-PCR (Figure 2C) and immunoblot (Figure 3) analysis demonstrated that the *gfp* tagged *merE* was successfully integrated into the genome of *Arabidopsis* plants and substantially transcribed into mRNA and then translated into the expected fusion proteins in the transgenic plants. Transgenic *Arabidopsis* plants expressing *merE* grew vigorously at rates similar to those of wild-type plants, without exhibiting notable symptoms of stress (Figure 5A). These results suggest that the integration of *merE* gene had no deleterious effects on the plant growth. The transgenic *Arabidopsis* expressing MerE accumulated significantly more Hg(II) and CH_3_Hg(I) than the wild-type *Arabidopsis* from the mercurial-containing medium (Figure 4). These results reveal that MerE is indeed functional as a broad-spectrum mercury transporter in transgenic *Arabidopsis*, and suggest that accelerated mercurials uptake into the plants mediated by MerE would provide one possible way for shortening the completion time of phytoremediation of mercury pollution.

Growth in a relatively higher concentration of mercurials and constitutive expression of mercury transport activity seem to be necessary for the plants applied in mercury remediation. The transgenic *Arabidopsis* displayed a relatively high level of Hg(II) and CH_3_Hg(I) resistance compared with the wild type (Figure 5). These results demonstrated that the toxic Hg(II) and CH_3_Hg(I) in the culture medium may have been transported into plant cells by the integrated *merE* and substantially inactivated in the cells by the physiological activity of the plant.

Phytoremediation is an effective and aesthetically pleasing technique for cleaning up soils contaminated with mercurials where excavation or bioremediation is not practical or possible (Heaton et al. 2003; Lyyra et al. 2007; Meagher and Heaton 2005). However, the technique is still in its infancy stage. This study showed that the expression of the bacterial mercury transporter MerE promoted the transport and accumulation of mercuric ions and methylmercury in transgenic *Arabidopsis*, which may be a useful method to facilitate the improvement of plants that could be applied to the phytoremediation of mercuric ions and methylmercury pollution. It is hoped that the efficiency of these newly-designed transgenic *Arabidopsis* plants will be validated in field experiments in the near future.

## Competing interests

The authors declare that they have no competing interests.

## Authors’ contributions

YS carried out the molecular studies, characterized transgenic plants, participated in analysis of mercury accumulation and resistant, and helped to prepare the manuscript. RN contributed valuable suggestions to the study. MHS carried out the cellular localization of GFP-MerE and supervised experimental design. HPH and TI supervised experimental design and revised the manuscript for submission. MK conceived and conducted the conception and design of the study and drafted the manuscript. All authors read and approved the final manuscript.
